# Taking a broader view of Three Gorges Dam

**DOI:** 10.1093/nsr/nwac032

**Published:** 2022-02-24

**Authors:** Emily H Stanley

**Affiliations:** Center for Limnology, University of Wisconsin–Madison, USA

As the world creeps forward toward a point of no return for global climate change, a wide range of strategies are being pursued to reduce greenhouse gas (GHG) emissions. Low GHG-producing energy alternatives to fossil fuels are at the center of these activities, and this includes hydropower development. Proponents of this alternative energy source emphasize its renewability, co-benefits provided by reservoirs and generation of electricity with little net release of GHGs to the atmosphere [1,2]. However, this latter argument has been questioned, as studies demonstrating often-high emissions of CO_2_, CH_4_ and N_2_O from hydropower reservoirs have accumulated [3]. And it has been suggested that the construction of Three Gorges Dam (TGD) may be one of these cases in which reservoir formation has led to greater GHG emissions [4].

In this issue, Ni *et al.* [5] provide the most comprehensive assessment to date of the Yangtze River's GHG dynamics to determine whether TGD has in fact substantially increased aquatic emissions. Assembly of a large dataset and use of machine learning tools allowed the authors to quantify GHG dynamics before and after impoundment to distinguish between ‘new’ emissions caused by damming and emissions that would occur naturally in the absence of the dam. This separation is essential for a robust assessment of the GHG consequences of dams, yet few studies have been able to do so [6].

While most studies of reservoir GHG emissions consider fluxes from reservoir surfaces and perhaps also the river immediately downstream from the dam where rapid degassing can occur, a second key strength of the work by Ni *et al.* is their recognition that the influence of TGD extends far beyond the reservoir and its immediate surroundings. Their approach is based on the logic that a full accounting of  GHG consequences of the dam should encompass the full sphere of the dam's influence. This means expanding the study extent to include the impounded reach as well as thousands of kilometers of the middle and lower Yangtze and delivery of gases to the East China Sea. This methodology led to the unexpected result that aquatic emissions have declined rather than increased since the dam went into operation.

The general lesson of this paper is that a larger whole-ecosystem view is needed to understand the effects of large dams. Expanding the spatial extent of study led to the revelation of declining GHG emissions from the Yangtze River. By itself, this conclusion may be viewed as a positive result regarding dam effects. However, expanding beyond this single process revealed fundamental and profound modifications of several other riverine processes following dam closure. As Ni *et al.* note, the dam has disrupted physical, chemical and biological conditions over large spatial and temporal scales ‘far beyond our previous understanding’. Although TGD is unique in its size, it is unlikely to be unique in the scale and scope of its influence; Ni *et al.* provide us with a reason to reconsider effects of large dams worldwide from larger and more expansive perspective.

***Conflict of interest statement*.** None declared.
